# Report on the Use of Telehealth in Early Intervention in Colorado: Strengths and Challenges with Telehealth as a Service Delivery Method

**DOI:** 10.5195/ijt.2019.6273

**Published:** 2019-06-12

**Authors:** BETH COLE, KATHERINE PICKARD, ARLENE STREDLER-BROWN

**Affiliations:** 1 EARLY INTERVENTION COLORADO, DENVER, CO, USA; 2 JFK PARTNERS, UNIVERSITY OF COLORADO SCHOOL OF MEDICINE, AURORA, CO, USA; 3 DEPARTMENT OF SPEECH, LANGUAGE, AND HEARING SCIENCES, UNIVERSITY OF COLORADO, BOULDER, CO, USA

**Keywords:** Early Intervention, Part C, Stakeholder Feedback, Telehealth

## Abstract

The use of telehealth as a service delivery method for early intervention (EI) is in its infancy and few studies have examined its use within the context of a statewide program. The focus of this report was to determine the factors that influence providers’ utilization of telehealth in Colorado’s Part C Early Intervention program (EI Colorado). This report presents information that was gathered through surveys sent to Part C program administrators, service coordinators, providers, and caregivers. Surveys were used to understand perceptions of telehealth, actual experiences with telehealth, and perceived benefits and challenges using this service delivery method. Follow-up focus groups were conducted with program administrators and family members to gather more nuanced information. Participants identified several benefits associated with telehealth including its flexibility, access to providers, and more family engagement. The primary barriers included access to high speed internet and the opinion that telehealth was not as effective as in-person treatment. The results in the report served to identify next steps in the implementation of telehealth in Colorado’s Part C EI program.

Access to the internet has steadily increased over the past decade (Meadan & Daczewitz, 2015), with 78.50% of families reporting that they have access to the internet with speeds fast enough to use video conferencing services. Increased access to the internet makes the use of telehealth, the delivery of health services via internet and video conferencing technology, a more viable and cost-effective service-delivery option for EI services (Little et al., 2018). A growing body of research examining the use of telehealth has demonstrated that this method of service delivery is a viable option for EI, either by itself or in combination with in-person visits (Baggett, et al., 2010; Cason, 2011; Little, Wallisch, Pope, & Dunn, 2018).

Many young children who have developmental delays or disabilities fail to receive EI services. This is particularly applicable to families who live in rural areas; these families are at increased risk of not receiving EI services and/or may not have access to appropriate specialists (Adams, & Tapia, 2013; Baharav & Reiser, 2010; Cason, 2011; Rosenberg, Zhang, & Robinson, 2008; Vismara, Young & Rogers, 2012).

Telehealth is also a way to overcome barriers faced by providers. Providers may find it difficult to deliver in-person therapy due to scheduling difficulties, illness, and/or challenging weather conditions.

Importantly, telehealth both necessitates and supports the use of family coaching strategies (Stredler-Brown, 2017). These strategies have been shown to increase family engagement and empowerment as families learn to apply what they have learned to their daily routines (Baharav & Reiser, 2010; Cason, 2011; Vismara et al., 2012). According to several research studies conducted with young children with Autism Spectrum Disorder, telehealth results in more active family engagement (Baggett, et al., 2010; Baharv & Reiser, 2010; Ingersoll, Straiton, Casagrande, & Pickard, 2016; Meadan & Daczewitz, 2015; Vismara et al., 2012), resulting in high levels of parent empowerment and self-efficacy, as well as positive child outcomes (Little et al., 2018; Vismara et al., 2012; Wainer & Ingersoll, 2015). According to Wainer and Ingersoll (2015), parents indicated that services delivered via telehealth were “acceptable, useable, and effective” (p. 3886).

Little research to date has examined the efficacy of EI services provided via telehealth when compared to those delivered in the home. Preliminary data from an ongoing telehealth study provides promising insight into the question of efficacy, demonstrating that young children who are deaf or hard of hearing (DHH) benefit equally when receiving weekly speech-language-listening intervention through in-person or telehealth delivery methods (Falcone et al., 2018). These findings parallel those from an earlier, small study by Blaiser, Behl, Callow-Heusser, and White, (2013) that compared the efficacy of telehealth services to those delivered in-home for another group of young children who are DHH. Blaiser et al., (2013) demonstrated that telehealth services were more efficacious than services delivered in-home, and that telehealth services resulted in significantly greater parent engagement in therapy sessions. In general, however, efficacy studies to date have been preliminary. There is also a paucity of research examining family and provider perceptions of the efficacy of telehealth. Importantly, no studies have been found to document these perceptions within a large, statewide system of care. This report intended to investigate the perceptions of stakeholders working in Colorado’s Part C system.

In 2015, EI Colorado investigated the use of telehealth as a way to address provider shortages. A task force was convened to examine factors to address these provider shortages. Committing to telehealth as a partial solution, EI Colorado made policy changes, wrote new procedures, systematically increased public awareness, provided training, and arranged billing for telehealth services. In Colorado, telehealth is paid by both private insurance, due to legislation that went into effect in 2017, as well as Medicaid. State and federal Part C funds may also be used to pay for telehealth visits. In 2017, EI Colorado launched four online training modules to prepare providers in the use of telehealth.

Despite the efforts of EI Colorado to support EI providers’ use of telehealth, utilization has been minimal. From July 2017 through July 2018, an average of 8,542 children per month were enrolled in EI Colorado (Early Intervention Colorado, 2018b). This is 3.78% of the total number of children, birth to three, in Colorado (OSEP, 2018). During the same time period, from July 2017 through September 2018, only 440 sessions were provided using telehealth as the service-delivery method. These sessions represent less than 0.1% of all EI services delivered to children in Colorado (Early Intervention Colorado, 2018a). Given the small number of providers using telehealth, EI Colorado decided to investigate perceptions of telehealth as a service delivery method.

This report presents results from surveys completed by key stakeholders including administrators of Part C regional programs, service coordinators, EI providers, and parents. A small number of focus groups were convened to provide additional information about stakeholders’ perceptions of telehealth. The surveys gathered information from individuals with and without telehealth experience. In this way, a variety of perspectives were available for consideration. This was deemed to be an effective way to understand attitudes that may impede the adoption of telehealth statewide. Due to the higher numbers of survey responses from service coordinators and providers, this report focuses on the responses from these two groups. In addition, focus groups were conducted with administrators to gather more nuanced information about the perceptions of telehealth that were reported in surveys. This report presents data from both the surveys and follow-up focus groups and addresses the following questions:

1. What are the perceived benefits of using telehealth?
2. What are the perceived barriers to using telehealth?
3. What suggestions do you have to improve the feasibility and acceptability of telehealth as a service delivery option?

## METHODS

Four surveys were developed by the original task force members convened by EI Colorado. The surveys targeted four distinct populations: (a) administrators in 20 regional Community Centered Boards (CCBs) responsible for Part C activities; (b) service coordinators working in CCBs; (c) providers serving children enrolled in Part C; and (d) parents of children receiving Part C services. In addition to the surveys, three focus groups were convened.

### PROCEDURES

Links to the online surveys were sent via email to local program administrators who were asked to complete the administrator survey and to send additional surveys to all providers, service coordinators, and families. Each survey focused on each group’s use and perceptions of telehealth within the EI system. Questions asked in the online survey included both open-ended questions and fixed questions. Comments were encouraged.

The open-ended questions included the following:

1. When do you typically use telehealth?
2. What do you like most about telehealth?
3. What do you like least about telehealth?

The fixed, multiple-choice questions addressed the following:

1. The percentage of children on a provider’s or service coordinator’s caseload who were receiving services via telehealth.
2. The reason(s) for using telehealth.
3. The effectiveness of telehealth (including an option for those who have not implemented telehealth)

Follow-up focus groups were convened to provide clarification and more nuanced information about the most prominent themes that were identified in the initial surveys. Focus group participants were recruited through e-mail and were not required to be using telehealth as a service-delivery method. Focus groups were conducted as semi-structured interviews in which broad questions were followed by more specific probes, as applicable. The structured questions included in the focus group interviews were as follows:

1. What makes telehealth easy to use?
2. What are the main challenges to using telehealth?
3. How does telehealth compare to in-person services?
4. Many individuals say that telehealth requires more targeted parent participation than in-person sessions. What do you think about that?
5. What aspects of the technology required for telehealth might be challenging for parents and providers?
6. What is your understanding of some of the logistical aspects of telehealth?
7. What resources does your agency have to support providers using telehealth? What resources would your agency need to make telehealth easier to use?

### PARTICIPANTS

Responses to the surveys were submitted by 112 providers, 39 service coordinators, eight program administrators, and two families. The surveys were anonymous. Based on comments that were made, it is presumed that participants responded from diverse geographic areas of Colorado, including both rural and urban areas. Specific demographic information was not requested as part of the surveys.

Based on minimal survey responses from program administrators and families, two focus groups were convened with program administrators, and one focus group was held with parents. Focus group participants were recruited by email and consisted of a sample of convenience, as the participants were the only ones to respond. Participants were predominantly white and all identified as female. The program administrators had experience with telehealth, but neither of the family participants was currently using telehealth for their services. Based on this, this report includes only the comments made by program administrators.

### DATA ANALYSIS

Three individuals analyzed the survey data. One individual was a staff member from EI Colorado who has been involved in Colorado’s telehealth effort from its inception. A second person is a speech-language pathologist at the University of Colorado-Boulder who is a co-investigator on an NIH-funded study about the use of telehealth with children who are deaf or hard of hearing (DHH). A third researcher, from the University of Colorado-Denver, brought her extensive experience in the use of telehealth. The authors’ experiences supported telehealth as a beneficial way to provide early intervention services.

Miles and Huberman’s (1994) approach to qualitative analysis was used to analyze survey and focus group data. In the first phase of data analysis, surveys and focus group interviews were de-identified and reviewed by the three investigators. Data were condensed into analyzable themes with concrete definitions (Miles & Huberman, 1994). Each investigator independently coded the data. Consensus coding was used to establish reliability when there was disagreement on specific themes. Analysis of final themes was facilitated by the use of Dedoose software. Primary themes and definitions are provided in Table 1.

## RESULTS

There were 112 responses from providers, with 80% of the respondents (*n*=90) reporting that none of the children on their caseload were receiving services via telehealth. Of the 46 providers who answered questions about the use of telehealth, 30 actually had some level of experience with it. The remaining 16 providers were interested in using telehealth, but had not yet started to use it. Since only 20% of providers (*n*=22) were currently using telehealth for EI visits, most of the responses reflected perceptions of telehealth rather than actual experiences using it.

Thirty-nine service coordinators responded to the initial survey. The vast majority of service coordinators, 97% (*n*=38), indicated that less than 25% of their caseload was receiving services via telehealth.

Eight administrators from the 20 local programs (40%) returned surveys. Only two families returned the survey.

Table 1 identifies the primary themes identified in the surveys, as well as the frequency with which these themes were mentioned by providers and service coordinators.

Using a frequency table in Dedoose, five themes emerged with the largest number of comments. They are: (a) flexibility; (b) provider shortages; (c) use of coaching practices; (d) technology barriers; and (e) attitudinal barriers.

The results that follow highlight specific examples of these five themes. Additional information was gathered from focus group respondents.

### FLEXIBILITY

When asked about the benefits of telehealth, providers and service coordinators consistently cited the flexibility provided by this delivery method. Flexibility was the most prevalent theme for both providers and service coordinators and was reflected in several different ways. Some mentioned the ability to have visits during a family’s typical routines, such as dinnertime, when it would be difficult for a provider to attend in person. One administrator said, “It (telehealth) adds the flexibility. They (the family) focus a lot more on dinner…they’ve been able to actually have the iPad at the dinner table and focus more on real life skills...”

Other providers reported that telehealth would allow them to have more visits during the day because drive time was reduced. An administrator noted that telehealth could be used to support transdisciplinary teams by saying, “I think you could have virtual teaming meetings to really have conversations about sharing of information and almost like a co-treatment kind of approach where we’re supporting one another.” Finally, many participants indicated that telehealth would allow them to deliver services in the event of illness or poor weather. For example, a service coordinator stated, “Families can still get EI services in the event that something prevents the provider doing an in-home visit, whatever the reason may be.”

### INCREASED ACCESS TO CARE FOR RURAL FAMILIES (PROVIDER SHORTAGES)

Another prominent theme from the surveys and focus groups was that telehealth provided access to providers and specialists for families living in rural areas where these specialists may not be available. An administrator from a rural area stated, “…one of the things that we would like to be able to do is offer the expertise that we might have in more of an urban area to some of the rural areas. So I see that there could be the potential of some sharing of expertise for those rural areas that do have challenges finding therapists.” Another administrator from a rural area in the southeastern part of the state stated, “…we’re very rural and I don’t have a lot of therapists actually in the area, and the ones that we do are on with the hospital or private practice. There is a non-compete clause in a lot of their contracts and I had to reach out to people in Lafayette and Denver just trying to get a therapist down in this area that will work with us (via telehealth).” Similarly, a provider stated, “This (telehealth) was used primarily because the families lived far away. We were able to have more sessions because the time needed for travel was eliminated. Extra sessions were helpful for the child and family.”

### SUPPORT FOR FAMILY COACHING PRACTICES

While the number of comments in the surveys regarding coaching were not as high as some of the other themes, coaching was a prominent theme in the focus groups. Service coordinators, providers, and program administrators indicated that telehealth models heighten family engagement and the use of coaching practices. Participants reported that using telehealth engages parents in their child’s sessions resulting in sessions being more aligned with the core principles of early intervention. An administrator stated that, “The therapists are really liking it (telehealth) because it gets the parents and the families more involved. Because it’s one-on-one, and they (parents) had to actually sit down and work with them (children). And then talking to the parents, they (providers) actually like that (telehealth sessions) because it doesn’t feel like someone was just coming in and working with their child while they (parents) just sit on the couch and watch.” Another administrator expressed that modeling and coaching are a huge benefit of telehealth because the provider cannot revert to a hands-on, clinical model. This administrator also reported that telehealth’s support of coaching gets at the true meaning of best practice in EI.

Despite the positive perceptions surrounding increased family engagement through telehealth, some providers and administrators noted that they were not sure if providers were adequately trained in the use of family coaching practices. One administrator stated, “I do think a barrier to that (coaching) is that there is not a class in their OT schooling that teaches them about providing family-centered modeling. So I think it almost starts from an educational place of when OTs are receiving their certification. They’re doing internships or practicums in more of those clinical settings, and so they are just not getting that experience that is needed to provide that coaching and modeling from the beginning.”

### TECHNOLOGY BARRIERS

Participants also spoke about barriers related to the use of internet technology and video conferencing platforms. One administrator stated, “…you have to spend quite a bit of time on the front end getting everything set up and testing and doing all of that and for people, the known (in-person therapy) is just more efficient or more comfortable than the unknown (telehealth)…” and “…families don’t always have the technology so that initial set up can be really challenging.” Other participants emphasized that the rural families who would benefit most from telehealth are also the ones who have limited access to internet connections. They noted that telehealth within the EI system currently needs to be delivered over a secure connection, which rules out more accessible video conferencing platforms that a family could access using cellular data from their phones. For example, a provider indicated, “I do not use it because most of my families do not have a computer with a secure internet connection.” An administrator similarly said, “So there are logistically some kinks and especially that secure connection seems to be the one that I hear time and time again from providers. Like, if we could just do it in FaceTime on our iPhones, it would be so much simpler.” Providers cited that, given their frequent driving, they often do not have their own private office from which they can provide secure telehealth services. One provider stated, “I was surprised to find that I had a hard time finding a place to do it (telehealth) myself…if it (holding the session) doesn’t work in my (the provider’s) own home, for example, because we’re in between children…you still have to have a place to be.”

### ATTITUDINAL BARRIERS

A handful of service coordinators and providers reported negative family attitudes toward the use of telehealth as a reason for its minimal use. Since 80% of providers and 97% of service coordinators who responded were not using telehealth, it could be speculated that most of these comments were based on preconceived ideas and not on actual experience. Some providers and service coordinators indicated that they perceived services delivered via telehealth to be less personal, and that was a reason not to use this method. One provider stated, “If we are able to provide the service in-home -- that seems to be the family’s preference.” A service coordinator stated, “I have found in most circumstances when I did recommend (telehealth), the family declined. Families are still wanting in-person sessions with providers. I think this will work better in some communities than others.”

## DISCUSSION

### PRIMARY FINDINGS

This report is one of the first to examine provider, administrator, and service coordinator perceptions regarding the use of telehealth services within a statewide EI program that both endorses and aims to increase the use of telehealth. Due to the very small number of family members who completed the survey and participated in the focus group, results would not be representative and were not included in this report. In general, findings from this report suggest that use of telehealth adds flexibility for providers and families. Providers and service coordinators indicated that telehealth allows them to increase the number of visits to a family, as well as the total number of families seen, if they are not spending as much time driving to families’ homes. Similarly, providers reported that they are able to have visits during non-traditional hours. Having therapy sessions at non-traditional hours, but during important daily routines (e.g., mealtime), provides families with tools in real-time to address challenges in a meaningful manner.

In addition to added flexibility, survey participants emphasized that using telehealth also supports a family coaching model, which is best practice for early intervention (McWilliam, 2016; Rush & Sheldon, 2011). Some of what was learned, however, is that not all providers have training in family coaching models and/or have limited experience coaching families, despite this being a critical aspect of EI services.

The information collected from all stakeholders illustrates the need to support providers so they understand the logistics of delivering services via telehealth, as well as the benefits of this service delivery method. To increase awareness of the logistics of telehealth, the state EI program could provide additional education to administrators, service coordinators and EI providers regarding its use.

A handful of service coordinators and providers stated that they felt telehealth was less effective, less personal, or that families would not like it. This finding is contradicted by emerging research suggesting that telehealth may be equally effective as in-person services (Blaiser, et al., 2013; Falcone et al., 2018), and reports that families have positive perceptions of telehealth (Pickard, Wainer, Bailey, & Ingersoll, 2016). By assuming limited efficacy and negative family attitudes about telehealth, providers and service coordinators may not be offering telehealth services to families who could benefit from it. Efforts to change these attitudes regarding telehealth will require a comprehensive plan.

### IMPLICATIONS

Since telehealth is still a new delivery method for EI services, there is a critical need for more research demonstrating the efficacy of this method and, particularly, a comparison of telehealth to in-home models of care. Clinical research in this area will help EI Colorado develop public awareness strategies that may help to alter the attitudes of families, providers, service coordinators and administrators so that they begin to trust that telehealth could be a viable and effective method for early intervention.

The findings from this report suggest that the flexibility of telehealth is of interest. The knowledge that telehealth supports a family coaching model needs greater emphasis. However, this emphasis may need to be paired with more explicit training for providers regarding how to provide family coaching. Increased comfort with the coaching model may lead to increased use of telehealth. It is anticipated that, one day, telehealth will become a common method of service delivery throughout Colorado’s EI system.

### LIMITATIONS

There are a few important limitations to consider about this report. First, the focus group results are drawn from a small number of participants from within a specific community setting. Therefore, the focus group results may not generalize to all areas of Colorado. Additionally, few family perspectives were gathered as part of this report. Although the small number of family perspectives that were collected were generally positive, they were gathered from families with limited experience with telehealth. Finally, the surveys did not ask for participants’ specific demographic data. Therefore, it is not possible to know the demographic make-up of participants, and whether or not participants were representative of all service coordinators, providers, and families within EI Colorado.

### NEXT STEPS

Based on the results from this report, providers need support managing the technology that is required for telehealth, such as the use of secure telehealth platforms. Technology management emerged as one of the primary structural barriers that interfered with the use of telehealth. Providers will also need concrete strategies to solve problems accessing adequate internet bandwidth in underserved, rural areas in Colorado.

In addition, research is needed that compares the child outcomes of EI sessions delivered via telehealth to those delivered in person or through a hybrid delivery model (i.e., some services provided in-person and some services provided through telehealth). Data from this type of research may provide the evidence supporting the effectiveness of telehealth. This finding, in turn, may help change providers’ and families’ willingness to try it.

Important next steps for EI Colorado include the need to support administrators, service coordinators, providers, and families in understanding the benefits that are already known about telehealth. This will require increased public awareness, including the use of family stories about the successful use of telehealth. In addition, EI Colorado can systematically disseminate existing reports describing positive perceptions of telehealth, as well as preliminary efficacy data about telehealth (Pickard, et al., 2016; Vismara et al., 2013; Wainer & Ingersoll, 2015). Receipt of this knowledge could help to influence and change attitudes about the use of telehealth. There is a large body of research demonstrating the important role that attitudes have on behavior change (Azjen, 1991). Future work by EI Colorado can be guided by the Theory of Planned Behavior to support attitudinal changes. The Theory of Planned Behavior suggests that a number of factors influence behavior change, including attitudes towards the behavior, self-efficacy in completing the behavior, and norms surrounding the behavior (Azjen, 1991).

Results from this report suggest that it will be important for EI Colorado to provide more training and support to providers on the use of coaching strategies in their work with families. When providers are confident in the use of coaching strategies, they may become more comfortable using telehealth. Once providers learn to use coaching practices, which are considered best practice in early intervention (Rush & Sheldon, 2011), they may also generalize its use to in-person sessions.

Finally, service coordinators and providers require instruction about the ways to introduce telehealth to a family. Practitioners reported that they selectively introduce telehealth to families, based upon their subjective perceptions of who will benefit from telehealth as well as like it. Unfortunately, this means that many families may not be aware that telehealth is a viable service-delivery option. EI Colorado takes the position that telehealth can be introduced to all families.

## Figures and Tables

**Table 1:** Themes and Frequency of Occurrence from Survey Data

Theme	Theme Definition	Frequency of Comments: Providers	Frequency of Comments: Service Coordinators
Efficacy	How well telehealth works compared to in-person sessions	2	5
Family Attitudes	Family choice and/or issues families voiced; includes comments described as concerns for families	24	26
Flexibility	Flexibility as a reason for using telehealth	69	42
Less Personal	Any comment that talks about telehealth as less “personal“ than in-person visits	24	16
Less Rapport	The perceived challenge establishing and/or building rapport with families when using telehealth	15	4
No Direct Work	Providers stating that telehealth prevents direct work with a child	22	7
No Modeling	Providers stating that telehealth does not allow modeling or demonstration of strategies	16	0
Other Provider/Service Coordinator Attitudes	Any comment that demonstrates a provider’s attitude regarding telehealth	27	13
Parent Coaching	The impact of telehealth on parent coaching and/or increased family involvement	31	9
Procedural Issues/Barriers	Paperwork required to begin telehealth sessions	13	14
Productivity	Any comment about telehealth impacting the productivity of therapy sessions	26	2
Provider Shortages	Any comment that implies telehealth may be a solution to provider shortages in rural areas and/or increases access to specialty providers	55	17
Technology Issues	Technology (e.g., internet connection, hardware, software) as a barrier to telehealth	57	30
Travel	Amount of travel that would be saved by a telehealth session	11	0
Weather	Telehealth as an option for inclement weather	14	9
